# Flap Donor Site Size Reduction with Substratum Horizontal Mattress Suture

**Published:** 2013-01

**Authors:** Mohammad Jalilimanesh

**Affiliations:** Department of Plastic and Reconstructive Surgery, Trauma and Burn Hospital, Yazd, Iran

**Keywords:** Skin graft, Donor site, Flap surgery

## Abstract

**BACKGROUND:**

Closure of donor site of the flap has special problems. Reduction of this site will decrease the morbidity of operation. In this study, we present our experience in donor site size reduction.

**METHODS:**

Between 2006 and 2008, 15 patients with skin and soft tissue defects underwent operation. In all patients, coverage of defect was performed with various flaps. Substratum horizontal mattress suture was used to reduce donor site dimensions. In all 15 patients, size of the flaps, the defect after the flap elevation and the scar size were measured.

**RESULTS:**

The mean size of the flap, the defect after flap elevation, and the scar after 3 months were 43.9 cm^2^, 69.4 cm^2^, and 32.2 cm^2^, respectively. There was 46.5% reduction in the donor site after using this suture.

**CONCLUSION:**

The substratum horizontal mattress suture was shown to de- crease the donor site dimensions and also its scar size in flap surgery. This suture is highly recommend in order to reduce

donor site dimensions.

## INTRODUCTION

The use of skin flap for soft tissue reconstruction is a common procedure in plastic surgery.^1^ Donor site closure is one of the major disadvantages of flaps and has a supplementary role in flap surgery.^2^ In small flaps, the donor site can be repaired primarily, but in moderate and large flaps, skin graft is necessary.^2^ Skin graft damages and another area of the body lead to a higher morbidity.^2^ Skin graft donor site may suffer from complications of fluid loss, excessive pain and prolonged period of healing, hypertrophic scaring and undesired pigmentation.^3^ The less the skin removal, it causes the less patient discomfort and also the better aesthetic results.^3^ There have been few studies on donor site management and also on donor site size reduction.^3^ This study attempts to present our experiences in reduction of donor site size using a new technique in 14 patients.

## MATERIALS AND METHODS

Between 2006 and 2008, 15 patients with soft tissue defects were treated using various flaps (Table 1). Eight patients with reverse island sural flap (RISF), 4 patients with abdominal flap, 2 patients with thoracic flap and 1 patient with radial forearm flap were enrolled. In all patients, the donor site was too large to be primarily closed. In all patients, the flap dimensions ranged from 4×6 to 9×10 Cm. The donor site was minimized by substratum horizontal mattress sutures. In all patients, the flap size, the skin defect after flap elevation and the scar size after 3 months were measured.

**Table 1:** Type of flap, flap size, defect size after flap elevation, scar size and reduction among 15 Patients

**Patients**	**Type of Flap**	**Flap size (cm)**	**Defect after flap elevation (cm)**	**Scar size after** **3 months (cm)**	**Reduction percent**
1	RISF	6×5	6.5×6.8	6.5×2.3	33.8
2	RISF	8×4	8.8×5.9	8.7×2.6	43.5
3	RISF	6×5	7.1×6.8	7×3.1	44.5
4	RISF	7×5	7.9×5.8	7.6×3.3	54.5
5	RISF	6×4	7×5.8	7×2.5	43
6	RISF	8×5	9×7.1	8.6×3.8	51
7	RISF	6×5	9.9×6.8	9.8×4.1	59.5
8	RISF	6×5	7.1×7.2	7×3.2	43.5
9	Abdominal flap	10×9	15.7×10.1	6.3×9.8	38.9
10	Abdominal flap	8×9	12.2×9.5	6.5×9.4	52.5
11	Abdominal flap	9×8	12.5×8.9	5.5×8.8	43.5
12	Abdominal flap	8×7	11.6×8.2	5.6×8.2	48.2
13	Thoracic flap	6×4	8.3×4.9	8.3×2.3	46.9
14	Thoracic flap	6×5	8.5×6.1	8.5×2.9	47.5
15	Radial forearm flap	8×6	8.5×6.5	8.2×3.1	46
Mean dimension		43.9 cm^2^	69.4 cm^2^	32.2 cm^2^	46.5

**Fig. 1:**
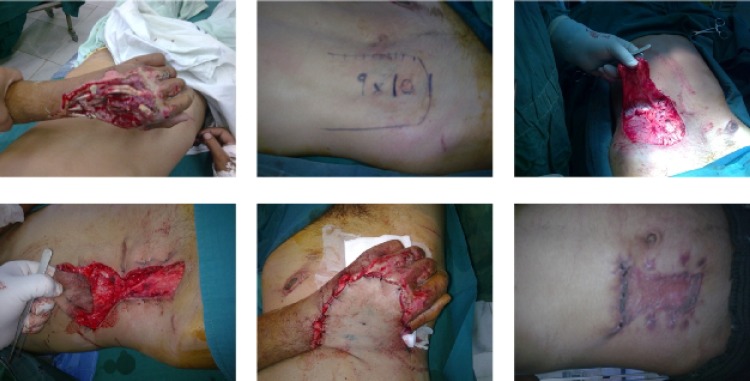
Above, left: Large hand soft tissue defect. Above, middle: Abdominal flap (10x9 cm). Above, right: Donor site after flap elevation (15.7x10 cm). Below, left: Donor site reduction whit three 0 nylon sutures. Below, middle: Complete coverage of defect. Below, right: Donor scar after three months (6.3x9.8 cm).

**Fig. 2:**
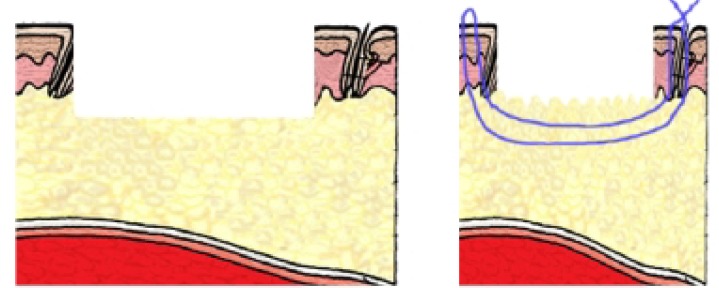
Left: Defect size of the skin and soft tissue. Right: Defect size after substratum suture.

**Fig. 3:**
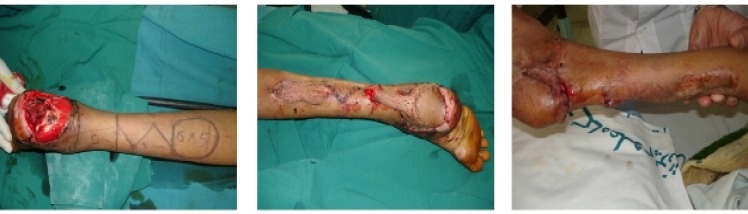
A: Large heel area soft tissue defect and RISF design (Flap size is 6x5 cm). Middle: Flap inset and donor site reduction with substratum horizontal matters suture. Right: Scar size at the time of pedicle division (After 3 months).

After elevation of the flap, the donor site reduction was carried out with 0 nylon suture (Figure 1). The needle was straightened and inserted from one border of the wound and then was moved under the soft tissue layer along the wound diameter and exited the skin border from other side. We tied the suture with moderate tension (Figure 2 and 3). For cover- age of the donor area, split thickness skin graft was harvested according to the new skin defect size after substratum horizontal mattress sutures. Skin graft dressing was removed after 5 days. The mattress sutures were removed three weeks after the operation.

## RESULTS

The donor site healed completely in all patients. The mean size of the flap, the mean size of the defect after flap elevation, and the mean scar dimensions after 3 months were 43.2 Cm^2^, 69.4 Cm^2^, and 32.2 Cm^2^, respectively. The overall reduction of donor area after using substratum horizontal mattress sutures was 46.5%.

## DISCUSSION

Flap surgery is an essential part of plastic and reconstructive surgery. Coverage of the donor site of the flaps has various complications.^2^

These include: pain, prolonged healing, hypertrophic scar and aesthetic problems.^2^

Primary repair of donor site is possible when the flap is small. In larger flaps, skin graft is necessary for coverage of the defect.^3^ The smaller the secondary donor site, the less morbidity and better aesthetic results ensues.^4^ Thin soft tissue layer over the suture facilitates complete taking of skin graft (Figure 3). If suture runs over the soft tissue, it will elevate skin graft from wound bed and increase the probability of graft failure.^4^^,^^5^ According to our experience and results, we suggest using this simple and useful suture for reduction of the donor site size during flap surgery.

## CONFLICT OF INTEREST

The authors declare no conflict of interest.

## Footenote

Please cite this paper as:

Jalilimanesh M. Flap Donor Site Size Reduction with Substratum Horizontal Mattress Suture. World J Plast Surg 2013;2(1): 14-17.

## References

[1] Ito O, Igawa HH, Suzuki S, Muneuchi G, Kawazoe T, Saso Y, Onodera M, Park S, Hata Y (2005). Evaluation of the donor site in patients who underwent reconstruction with a free radial forearm flap. J Reconstr Micro surg.

[2] Emerick KS, Deschler DG (2007). Incidence of donor site skin graft loss requiring surgical intervention with the radial forearm flap. Head Neck.

[3] Han SK, Yoon TH, Kim JB, Kim WK (2007). Dermis graft for wound coverage. Plast Reconstr Surg.

[4] Karimi A, Mahy P, Reychler H (2007). Closure of radial forearm free flap donor site defect with a local meshed full-thickness skin graft: A retrospective study of an original technique. J Craniomaxillofac Surg.

[5] Akan M, Yildirim S, Misirliogu A, Ulusoy G, Akoz T, Avci G (2003). An alternative method to minimize pain in the split-thickness graft donor site. Plast Reconstr Surg.

